# Percutaneous vertebroplasty *versus* percutaneous kyphoplasty in elderly patients with osteoporotic vertebral compression fractures: prospective controlled study

**DOI:** 10.1093/bjsopen/zrad162

**Published:** 2024-01-29

**Authors:** Qiang Wang, Junchuan Liu, Quan Ji, Yudian Qiu, Nan Min, Lin Wang, Yawen Zhang

**Affiliations:** Department of Orthopedics, Beijing Hospital, National Center of Gerontology, Institute of Geriatric Medicine, Chinese Academy of Medical Sciences, Beijing, China; Department of Orthopedics, Beijing Hospital, National Center of Gerontology, Institute of Geriatric Medicine, Chinese Academy of Medical Sciences, Beijing, China; Department of Orthopedics, Beijing Hospital, National Center of Gerontology, Institute of Geriatric Medicine, Chinese Academy of Medical Sciences, Beijing, China; Department of Orthopedics, Beijing Hospital, National Center of Gerontology, Institute of Geriatric Medicine, Chinese Academy of Medical Sciences, Beijing, China; Department of Orthopedics, Beijing Hospital, National Center of Gerontology, Institute of Geriatric Medicine, Chinese Academy of Medical Sciences, Beijing, China; Department of Orthopedics, Beijing Hospital, National Center of Gerontology, Institute of Geriatric Medicine, Chinese Academy of Medical Sciences, Beijing, China; Department of Orthopedics, Beijing Hospital, National Center of Gerontology, Institute of Geriatric Medicine, Chinese Academy of Medical Sciences, Beijing, China

## Introduction

Vertebral compression fractures are a common complication of osteoporosis with increasing global burden due to better life expectancies. The incidence of osteoporotic vertebral compression fractures (OVCFs) increases with age^1^ and the incidence of the disease in older adults is as high as 30–50%^2^. If the treatment of vertebral body compression fractures is not timely and standardized, it can significantly impact the quality of life of patients. In 1987, the first report of successful percutaneous vertebroplasty (PVP) for the treatment of vertebral haemangiomas was reported by Galibert^3^. PVP has been gradually promoted and widely used in the treatment of vertebral osteoporotic compression. Percutaneous kyphoplasty (PKP) adds an expandable balloon to further restore the original anatomical shape of the fractured vertebra. There are few prospective studies, but many retrospective studies comparing PVP and PKP in the treatment of OVCFs. The conclusions of existing randomized controlled clinical studies are conflicting^4,5^. The long-term benefit and safety in terms of postoperative quality of life have not been clearly demonstrated, and further prospective randomized studies and reporting standards are needed.

The aim of this study was to evaluate the efficacy, safety, and long-term quality of life improvement of PVP and PKP in elderly patients with severe thoracolumbar OVCFs.

## Methods

### Study design and participants

The study was performed in accordance with the Declaration of Helsinki, and the protocol was approved by the Ethics Committee of Beijing Hospital (2019BJYYEC-010-06). This clinical trial is registered in the Chinese clinical trial registry, registration number: ChiCTR1900021960. See *Text S1* for further information about the inclusion and exclusion criteria and sample size estimation.

Inclusion criteria: patients with osteoporosis over 60 years old, with vertebral body compression fractures caused by minimal trauma (falling on the ground) or no clear history of trauma, and imaging confirmed new vertebral body compression fracture, and the anterior height loss of the vertebral body was greater than one-third. Exclusion criteria: spinal compression fractures with signs of nerve compression requiring surgery for spinal canal decompression and internal fixation; pathological compression fractures caused by metastasis; coagulopathy; patients with local skin infection at the puncture site or systemic infectious diseases; and those who cannot be followed up as scheduled after treatment.

**Fig. 1:**
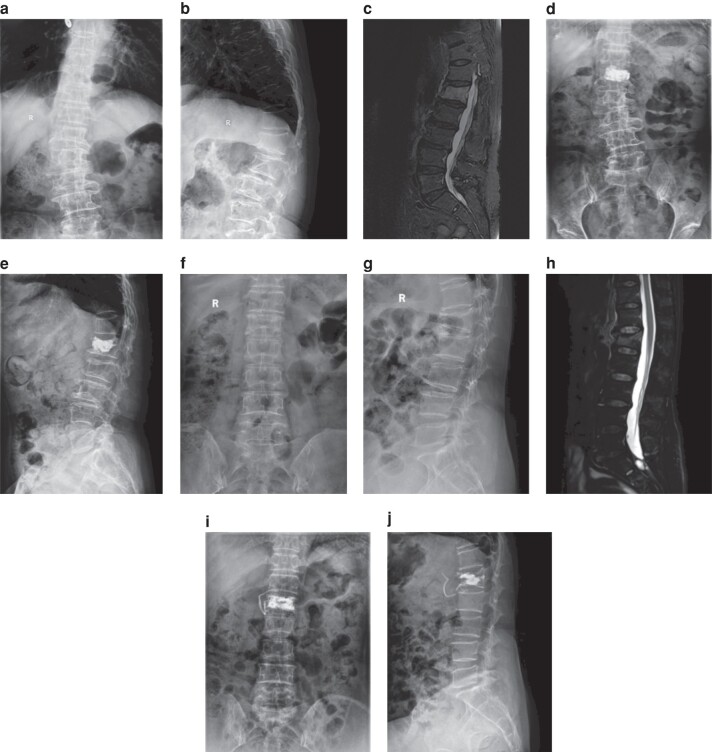
Percutaneous vertebroplasty (a-e) and percutaneous kyphoplasty (f-j) Percutaneous vertebroplasty: **a** AP thoracolumbar X-ray before surgery. **b** Lateral thoracolumbar X-ray before surgery. **c** Sagittal thoracolumbar MRI before surgery. **d** AP thoracolumbar X-ray post-PVP surgery. **e** Lateral thoracolumbar X-ray post-PVP surgery. AP, anterior-posterior; PVP, percutaneous vertebroplasty. Percutaneous kyphoplasty: **f** AP thoracolumbar X-ray before surgery. **g** Lateral thoracolumbar X-ray before surgery. **h** Sagittal thoracolumbar MRI before surgery. **i** AP thoracolumbar X-ray post-PKP surgery. **j** Lateral thoracolumbar X-ray post-PKP surgery. AP, anterior-posterior; PKP, percutaneous kyphoplasty; R, right.

Randomization was performed based on SAS software V9.4 to generate a random number table. A sample size of 58 patients in each group was chosen to provide 80% power to detect difference in levels of vertebral height recovery at a two-tailed-jα-error level of 0.05. Patients were randomly assigned to receive vertebroplasty (PVP group, *n* = 58, *Fig. 1*) or to accept kyphoplasty (PKP group, *n* = 58, *Fig. 1*). There was continuous enrolment of 116 patients who underwent vertebral compression fractures with a diagnosis of OVCF in our department and maintained vertebral augmentation therapy (PVP or PKP).

### Surgical management

All patients underwent PKP or PVP surgery within 2 days of admission. During this period, simple analgesia and bed rest were encouraged. See *Text S1* for further information.

### Outcome

The outcome was to compare the efficacy, safety, and long-term quality of life improvement of PVP and PKP. See *Text S1* for specific outcomes.

## Results

A total of 116 patients were recruited to the study (58 in the PVP group and 58 in the PKP group). One in the PVP group and three in the PKP group did not complete the whole trial. Demographic data in both groups were similar (*Table 1*). There was no statistical difference between the two groups in terms of age, gender, height, weight, BMI, preoperative anterior and posterior vertebral edge heights (*P* > 0.05, *Table 1*).

**Table 1:** Baseline characteristics and postoperative outcomes

Variables	PVP (*n* = 58)	PKP (*n* = 58)	*P*
Age (years), mean(s.d.)	73.2(5.4)	72.8(6.1)	0.686
**Sex**			0.103
Male	8 (8.9)	15 (22.9)	
Female	50 (91.1)	43 (77.1)	
Height (m), mean(s.d.)	160.2(7.0)	161.7(7.3)	0.267
Weight (kg), mean(s.d.)	63.1(9.7)	63.2(10.9)	0.928
BMI (kg/m^2^), mean(s.d.)	24.5(3.2)	24.1(3.3)	0.458
**Vertebral height (cm)**			
Anterior edge, mean(s.d.)	18.3(3.9)	18.2(3.5)	0.919
Posterior edge, mean(s.d.)	30.7(5.0)	30.8(5.0)	0.886

	PVP (*n* = 57)	PKP (*n* = 55)	*P*
**Complications**			
Leakage	10 (17.5)	18 (32.7)	0.064
Paravertebral veins	3 (30.0)	6 (33.3)	
Surrounding tissues	4 (40.0)	4 (22.2)	
Spinal canal	0 (0.0)	0 (0.0)	
Intradiscal	3 (30.0)	8 (44.4)	
Re-fracture	2 (3.5)	2 (3.6)	1.000
**Anterior edge height (cm)**			
Preoperation, mean(s.d.)	18.3(3.9)	18.2(3.5)	0.825
Postoperation, mean(s.d.)	22.7(4.2)	23.0(4.3)	0.714
**Price (Euros)**	4712	5969	

Values are *n* (%) unless otherwise stated. PKP, percutaneous kyphoplasty; PVP, percutaneous vertebroplasty.

Of the 112 people who completed the study, one patient in the PVP group did not complete the full 1-year follow-up and a total of three patients in the PKP group did not complete the full 1-year follow-up.

Cement leakage occurred in 10 patients in the PVP group, and 18 in the PKP group (*P* > 0.05, *Table 1*). At 1-year follow-up, two patients in the PVP group and two patients in the PKP group had suffered re-fracture (3.5% *versus* 3.6%) (*P* > 0.05, *Table 1*).

There were no differences in preoperation or postoperation anterior edge height between the two groups (*P* > 0.05, *Table 1*). Compared with preoperative data, the anterior edge height was improved with significant differences after surgery in both groups (*P* < 0.01, *Table 1*). According to the current total high-value consumables cost of a single vertebral body surgery segment in our hospital, the cost of PKP (€5969) is higher than that of PVP (€4712), according to the maximum amount paid by Chinese medical insurance Diagnosis Related Groups (DRG).

The preoperative and post-preoperative visual analogue scale (VAS) scores of patients in different groups are displayed in *Fig. S1*. The preoperative and postoperative SF-36 scores of patients in each group are displayed in *Fig. S2*. See *Text S1* for detailed statistical information.

## Discussion

Cement leakage is the most common complication in PKP and PVP. A review by Garfin *et al*. found that 34 to 64% of vertebral augmentation treatments involved cement leaks^6^. This study found that the incidence of cement leakage in practice was approximately 25% (28 of 112); there was no statistical difference in the incidence of this complication between the two groups. Local leakage of polymethyl methacrylate is frequent, but in most cases does not produce any symptoms^7,8^. Similarly, no related adverse events such as pulmonary embolism and spinal cord injury were found in our cases. Vertebral fractures can predict future vertebral fractures and other osteoporotic fractures. Among women with pre-existing vertebral fractures, the risk of a subsequent fracture was approximately four times higher than in women without a history of fracture, and this risk increased with the number of prior vertebral fractures^9^. This study demonstrated that female groups are at high risk of OVCF. A total of four patients experienced re-fracture (three at the non-contiguous spine level and one hip fracture), the incidence of re-fracture was 3.5%. There was no statistical significance between the two groups. In terms of restoring vertebral body height, both PVP and PKP performed well. Compared with preoperative assessment, the height of the anterior edge of the vertebral body increased by 4.4 and 4.8 cm respectively. Cost-effectiveness is a consideration for treatment decision. China's current medical resources are in short supply with high demand. Considering economic factors, the single vertebral segment high-value consumables price of PVP is €4712, which is €1257 less than that of PKP.

Existing studies have shown that these two operations can both achieve good clinical outcomes^10–12^. Nakano *et al.* found that the mean improvement in VAS scores in the PVP group and non-surgical (NS) group at 12 months was 91.6% and 73.6% better than baseline, respectively. There was also a significant difference in the amount of analgesia required between the two groups^13^. A total of 300 patients with 5- to 6-week-old OVCF were enrolled by the Fracture Reduction Evaluation Study, randomized to either PKP (*n* = 149) or NS (*n* = 151). The primary outcome was the difference in change in SF-36 score from baseline to 1 month between the PKP and NS groups, which was found to be significantly better in PKP-treated patients^14^. However, there are few prospective randomized controlled trials comparing the safety and long-term efficacy of PVP and PKP in China. There was no statistically significant difference in the VAS score between the two groups at the time point of 1 year after surgery. This suggests that the long-term analgesic effect of PVP is equivalent to the effect of PKP, which is consistent with previous research^15^. The SF-36 Health Survey Short Form is based on the Medical Outcomes Research Scale developed in 1988 and can evaluate overall health. In this study, both treatments significantly reduced the VAS score and improved the patients’ SF36 scores during long-term follow-up.

There are limitations to this study. The majority of the patients were female, in keeping with the higher incidence of osteoporosis in females. Multiple different surgeons performed the procedures. In addition, due to the impact of the coronavirus disease 2019 (COVID-19) pandemic, many patients spent more time at home, with a reduction in outings, which may have impacted the results of this study.

In this study, both PVP and PKP significantly relieved pain in patients with OVCFs, and improved restoration of vertebral body height and patients’ quality of life. Large-scale, multi-centre studies are still needed to further confirm the results.

## Supplementary Material

zrad162_Supplementary_Data

## Data Availability

The data sets used and analysed during the present study are available from the corresponding author upon reasonable request.
