# Supplementary material for: Percutaneous vertebroplasty versus percutaneous kyphoplasty in elderly patients with osteoporotic vertebral compression fractures: prospective controlled study
Source: BJS Open. 2024 Jan 29;8(1):zrad162. doi: 10.1093/bjsopen/zrad162 (PMC10823769; doi:10.1093/bjsopen/zrad162)

**PVP vs PKP in elderly patients with osteoporotic vertebral compression fractures: Prospective controlled study**

Qiang Wang, Junchuan Liu, Quan Ji, Yudian Qiu, Nan Min, Lin Wang, Yawen Zhang*

Department of Orthopedics, Beijing Hospital, National Center of Gerontology, Institute of Geriatric Medicine, Chinese Academy of Medical Sciences, Beijing, China

*Correspondence. Yawen Zhang, Email: [dr.zhangyawen@pku.edu.cn](mailto:dr.zhangyawen@pku.edu.cn);

**Supplementary Materials – Index**

**Supplementary Text, Figures**

**Supplementary Text S1** page 2 - 9

**Figure S1 –Change trend of VAS scores of patients in two groups before and after surgery** page 10

**Figure S2 –Change trend of SF-36 scores of patients in two groups before and after surgery** page 11

**Supplementary Text S1**

**Methods**

**Study Design and Participants**

The study was performed in accordance with the Declaration of Helsinki, and the protocol was approved by the Ethics Committee of Beijing Hospital (2019BJYYEC-010-06). Written, informed consent was obtained from all participants. This clinical trial is registered in the Chinese Clinical Trial Registry (ChiCTR1900021960).

Inclusion criteria: (1) Elderly people with osteoporosis over 60 years old, with vertebral body compression fractures caused by minor trauma (falling on the ground) or no clear trauma history; (2) The imaging examination confirmed that it was a fresh vertebral body compression fracture, and the anterior height loss of the vertebral body was greater than 1/3.We continuously enrolled 116patients (23 males and 93 females) (73 ± 5.7 years) who underwent vertebral compression fractures with a diagnosis of OVCF in our department and maintained vertebral augmentation therapy (PVP or PKP). Exclusion criteria are as follows: (1) Spinal compression fractures with signs of nerve compression requiring incision surgery for spinal canal decompression and internal fixation; (2) Pathological compression fractures caused by spinal metastatic tumors; (3) Patients with severe coagulation dysfunction; (4) Patients with local skin infection at the puncture site or systemic infectious diseases; (5) Those who cannot be followed up as scheduled after treatment.

Randomization was performed based on SAS software to generate random number table. A sample size of 58 patients in each group was chosen to provide 80% power to detect difference in levels of vertebral height recovery at a 2-tailed-α-error level of 0.05. Patients were randomly assigned to receive vertebroplasty (PVP group, n=58) or to accept the kyphoplasty (PKP group, n=58). A total of 1 patient in the PVP group did not complete the full 1-year follow-up, a total of 3 patients in the PKP group did not complete the full 1-year follow-up.

**Surgical management**

The operations of all patients were completed by surgeons with the title of deputy director or above in our hospital. All the patients received X-ray, computed tomography (CT), and magnetic resonance imaging (MRI) scans for accurate diagnosis of fresh fractures.

**PVP**: The patient was placed in a prone position with the abdomen slightly elevated, and after traction reduction, 2 Kirschner wires were fixed on the patient's lower back by the crisscross method, and the target position of the injured vertebra was positioned with a C-arm X-ray machine. The pedicle attachment of the local infiltration anesthesia machine was used for anesthesia. The puncture needle was used for routine puncture, and the abduction angle of the puncture needle was controlled to make the puncture needle reach the center of the vertebral body. Fluoroscopy showed that the puncture needle passed through the pedicle and reached the anterior 1/3 of the injured vertebra, and then stopped the needle insertion. After the working channel was established, the prepared High-viscosity bone cement was slowly injected into the vertebral body through a bolus injector. During the injection of bone cement, a C-arm X-ray machine was used for fluoroscopic observation to prevent leakage of bone cement. After the needle was withdrawn, the skin was routinely sterilized and a sterile dressing was applied to end the operation without sutures.

**PKP:** The methods of preoperative preparation and working channel establishment in the PKP group were the same as those in the PVP group. After the working channel was established, a balloon dilator was placed, and the injured vertebra was expanded routinely. The expansion was satisfactory under the fluoroscopic observation of the C-arm X-ray machine. After keeping the pressure stable for 3 min, the balloon dilator was removed and the prepared bone cement was injected into the injured vertebra. In the process of bone cement injection, a C-arm X-ray machine was used for fluoroscopic observation to prevent leakage of bone cement. After that, other operations were the same as the PVP group.

All patients underwent PKP or PVP surgery within 2 days after admission. During this period, simple analgesia and bed rest were encouraged, and patients were allowed to move freely on the first postoperative day, with regular postoperative anti-osteoporotic therapy. According to the latest guidelines, a patient with a vertebral fragile fracture can be diagnosed with osteoporosis and requires regular anti-osteoporotic treatment [1].

**Outcome**

Key metrics for research evaluation:

1) The main evaluation indicators from the perspective of patients' feelings are VAS score and SF36 score, both of which evaluate the improvement of fracture symptoms after surgery in terms of pain and quality of life.

2) The main indicators evaluated from the doctor's point of view are: X-ray measurement of the height of the anterior edge of the fractured vertebral body, the leakage rate of bone cement and re-fracture (fragile fracture) within 1-year.

3) The main indicator evaluated from the perspective of health economics is the total cost of fracture treatment.

**Statistical Analysis**

Age, gender, and other baseline factors were compared between the groups using independent sample T test method and chi-square analysis. The variation characteristics of VAS and SF36 in different groups and at different time points were analyzed by two-factor repeated measures analysis of variance. First, the Shapiro-Wilk test was performed to test whether the data in each group conformed to the normal distribution, and then it was judged whether there was an interaction between the two factors of different groups and different time points. If there is no interaction, the main effect calculation results are obtained directly. All data were subjected to split parallel one-way repeated measures ANOVA if there was an interaction. Before judging whether there is an interaction between the two factors, the Mauchly sphericity test is firstly performed to judge whether the spherical assumption is satisfied; when the spherical assumption is not satisfied, the Greenhouse & Geisser method is used for correction. In all analyses, P-values <0.05 were considered statistically significant.

**Results**

This prospective study included 116 patients, 23 males and 93 females, with a mean surgical age of 73. Of these 116 patients, there were 58 in the PVP group and 58 in the PKP group. 1 in the PVP group and 3 in the PKP group did not complete the whole trial. Demographic data in both groups were similar (Table 1). There was no statistical difference between the two groups in terms of age, gender, height, weight, BMI (P>0.05, Table 1). Similarly, there was no statistical difference in the preoperative anterior and posterior edge heights of vertebral between the two groups of patients (P>0.05, Table 1).

Of the 112 people who completed the trial. In the PVP group, cement leakage occurred in 10 patients, while the PKP group had 18 cases of cement leakage. We have categorized the cases according to the common leakage types [2, 3]. In the PVP group, there are 3 leakage cases occurred in paravertebral veins, 4 leakage cases occurred in surrounding tissues, no leakage case occurred in spinal canal and 3 patients occurred Intradiscal leakage. In the PKP group, there are 6 leakage cases occurred in paravertebral veins, 4 leakage cases occurred in surrounding tissues, no leakage case occurred in spinal canal and 8 patients occurred intra Intradiscal leakage. As for rate of cement leakage, the PVP group was better than PKP group (17.5% vs. 32.7%). However, there was no significant difference in the cement leakage rate between the two groups (P>0.05, Table 2)

By the end of the one-year follow-up, two patients in the PVP group and two patients in the PKP group had suffered re-fracture (3.5% vs. 3.6%). There was no statistical significance between the two groups (P>0.05, Table 2).

There were no differences in pre-operation or post-operation anterior edge height between the two groups (P>0.05, Table 2). Compared with preoperative data, the anterior edge height was improved with significant differences after surgery in both two groups (P<0.01, Table 2). According to the current total high-value consumables cost of a single vertebral body surgery segment in our hospital, the cost of PKP (5969 euros) is higher than that of PVP (4712 euros), according to the maximum amount paid by Chinese medical insurance Diagnosis Related Groups (DRG).

Specifically, the preoperative VAS score of patients in the PVP group was 7.7 ± 1.4 points, which decreased to 2.6 ± 1.6 points at 1 day after operation, 1.8 ± 1.3 points at 1 months after operation, 1.5 ± 1.3 points at 3 months after operation, 1.3 ± 1.3 points at 6 months after operation and 0.9 ± 1.1 points at 1 year after operation. In the PKP group, the preoperative VAS score was 7.6 ± 1.9 points, which decreased to 2.6 ± 1.6 points at 1 day after operation, 2.0 ± 1.5 points at 1 months after operation, 1.6 ± 1.2 points at 3 months after operation, 1.0 ± 1.1 points at 6 months after operation and 1.0 ± 1.1 points at 1 year after operation. (Figure S1). The results of the Shapiro-Wilk test showed that the VAS score data in different groups and at different time points conformed to a normal distribution. The results of two-factor repeated measures ANOVA showed that there was no interaction between different groups and different time nodes (F=0.683, P>0.05). The main effect of group on VAS score was not statistically significant, (F<0.001, P>0.05). The main effect of time factor on VAS score was statistically significant, (F=502.789, P<0.01). The Bonferroni test was used for pairwise comparison, and there was a statistically significant difference in the VAS score between before surgery and 1 day, 1, 3, 6, 12 months after surgery(P<0.05). Differences in VAS scores between 3 months after surgery and 1 and 6 months after surgery were not statistically significant (P>0.05). Differences in VAS scores between 6 months after surgery and 12 months after surgery were not statistically significant (P>0.05).

Specifically, the preoperative SF-36 scores of patients in the PVP group was 332.4 ± 123.1 points, which increased to 502.9 ± 122.8 points at 1 months after operation, 563.8 ± 110.8 points at 3 months after operation, 591.5 ± 108.7 points at 6 months after operation and 623.6 ± 108.5 points at 1 year after operation. In the PKP group, the preoperative SF-36 scores was 361.6 ± 120.0 points, which increased to 515.2 ± 105.3 points at 1 months after operation, 595.0 ± 105.8 points at 3 months after operation, 605.8 ± 115.9 points at 6 months after operation and 619.6 ± 102.5 points at 1 year after operation (Figure S2). The results of the Shapiro-Wilk test showed that the SF-36 scores data in different groups and at different time points conformed to a normal distribution. The results of two-factor repeated measures ANOVA showed that there was no interaction between different groups and different time nodes (F=0.797, P>0.05). The main effect of group on SF scores was not statistically significant, (F=1.245, P>0.05). The main effect of time factor on SF scores was statistically significant, (F=192.115, P<0.05). The Bonferroni test was used for pairwise comparison, and there was a statistically significant difference in the SF-36 scores between before surgery and 1, 3, 6, 12 months after surgery (P<0.05). Differences in SF-36 scores between 6 months after surgery and 3, 12 months after surgery were not statistically significant (P>0.05).

**Discussion**

The number of elderly people in China is increasing rapidly. By the end of 2020, there were about 191 million people aged 65 and above in our country, accounting for 13.5% of the total population; By 2035, there will be more than 300 million people aged 65 and over, climbing to around 380 million by 2050, accounting for 27.9% of the total population [4]. With the aging of the global social population, the incidence of osteoporosis is gradually increasing, and osteoporosis has attracted more and more attention. Vertebral compression fractures caused by osteoporosis are the most common complication. The incidence of osteoporotic vertebral compression fractures (OVCFs) increases with age [5]. A review of the literature suggests that the incidence of the disease in older adults is as high as 30-50% [6]. If the treatment of vertebral body compression fractures is not timely and standardized, it will seriously affect the quality of life of patients.

With the aging of our society, the number of osteoporosis patients is increasing year by year. Age and osteoporosis reduce the mechanical strength of bone, reduce bone mass, and affect the regulation of biological factors important for healing[7]. Vertebral compression fracture is one of the most common complications in patients with osteoporosis[8]. One-third of patients with vertebral compression fractures have intractable pain[9]. Pain can further cause loss of appetite, falling asleep with difficulty and activity limited in the elderly. Traditional conservative bed rest treatment will further aggravate acute bone loss, resulting in further loss of bone mass in patients, aggravating the degree of pain and the risk of refracture[10]. Prolonged bed rest may exacerbate underlying disease states and may lead to rapid dysregulation, lung damage, and increased mortality[11]. In 1987, Gailbert et al. described cement augmentation of vertebral as an effective treatment for vertebral hemangiomas[12]. Now, vertebral cement augmentation procedures are widely used for treating osteoporotic vertebral compression fractures when non-surgical treatment fail. It includes percutaneous vertebroplasty (PVP) and percutaneous kyphoplasty (PKP) which are two commonly recommend surgical methods for vertebral compression fractures based on the latest evidence-based medical literature[13, 14]. Vertebral augmentation has many advantages such as definite curative effect, simple operation, less trauma, safe operation, and less complications, and has become an important method for the treatment of OVCFs. With a prospective, comparative, and randomized clinical study design, we look forward to finding the best treatment plan for OVCFs and provide reference for the clinical treatment of OVCF.

Cement leakage is the most common complication in PKP and PVP. A review by Garfin et al. found that 34 to 64% of vertebral augmentation treatments involved cement leaks[15]. Our study found that the incidence of cement leakage in practice was approximately 25% (28/112), there was no statistical difference in the incidence of this complication between the two groups. Local leakage of PMMA is frequent, but in most cases does not produce any symptoms[16, 17]. Similarly, no related adverse events such as pulmonary embolism and spinal cord injury were found in our cases. Patients with leakage were asymptomatic. Vertebral fractures can predict future vertebral fractures and other osteoporotic fractures. Among women with pre-existing vertebral fractures, the risk of a subsequent fracture was approximately four times higher than in women without a history of fracture, and this risk increased with the number of prior vertebral fractures[18]. It can also be found from our research that female groups are the high-risk group of OVCF. We studied the re-fracture (fragile fracture) of patients within one year, A total of 4 patients occurred re-fractures, the incidence of re-fracture is 3.5%, there was no statistical significance between the two groups. In terms of restoring vertebral body height, both PVP and PKP performed well. Compared with preoperative, the height of the anterior edge of vertebral body increased by 4.4 cm and 4.8 cm, respectively. In clinical decision-making and management, the clinical effects of interventions should be considered on the one hand, and their costs should also be considered on the other hand. The economic situation is always an important factor affecting the treatment decision, especially patients with ordinary economic conditions often choose low-cost treatment methods or even give up treatment. China's current medical resources are in short supply and the demand is huge. Consider economic factors, the single vertebral segment high-value consumables price of PVP is 4712 euros, 1257 euros less than that of PKP.

Existing studies have shown that these two operations both can achieve good clinical outcomes[19, 20, 21], Nakano found that compared with the non-surgical (NS) group, the mean improvement in VAS scores in the PVP group at 12 months was 91.6% and 73.6% better than baseline, respectively. There was also a significant difference in the amount of analgesic between the two groups[22]. 300 patients with 5 to 6-weeks old OVCF were enrolled by the Fracture Reduction Evaluation Study, randomized to either PKP (n=149) or NS (n=151). The primary outcome was the difference in change in SF-36 score from baseline to 1 month between the PKP and NS groups, which was found to be significantly better in PKP-treated patients[23]. However, there are few prospective randomized controlled trials comparing the safety and long-term efficacy of PVP and PKP in China. In our study, both the PVP and PKP groups showed the ability to significantly reduce the total VAS score. The VAS score in the PVP group reduced from 7.7 ± 1.4 points preoperatively to 0.9 ± 1.1 points at 1 year after operation, and declined from 7.6 ± 1.9 preoperatively to 1.0 ± 1.1 points at the last follow-up in the PKP group. There was a statistically significant difference in the VAS score between the two groups at the time point of 1 year after surgery. This suggests that the long-term analgesic effect of PVP is equivalent to the effect of PKP, which is consistent with previous research result [24]. The SF-36 Health Survey Short Form is based on the Medical Outcomes Research Scale developed in 1988 and developed by the Boston Health Research Institute in the United States. This score can evaluate the overall health of the human body. In our study, both the PVP and PKP groups showed the ability to significantly reduce the total VAS score. In our study, both the PVP and PKP groups significantly improved the patients' SF36 scores during long-term follow-up. The patient's quality of life has been continuously improved, compared with preoperative.

The sex ratio of patients enrolled in this study was severely disproportionate, which may have biased the results of this study. Multiple surgical surgeons performed these procedures, which was also a confounding factor in this study. In addition, due to the impact of the Coronavirus Disease 2019 (COVID-19) Pandemic, many patients spend most of the time at home, significant reduction in outings, which had a certain impact on the results of this study. Large-scale, multi-center studies are still needed to further confirm this result.

**Conclusion**

In this study, both PVP and PKP significantly relieved pain in patients with OVCFs, improved restoration of vertebral body height and patients' quality of life. And PVP is equivalent to PKP in terms of cement leakage rate, refracture rate, long-term analgesia and quality of life. The PVP was more advantageous in terms of health economics. We hope that this study can provide a certain reference value for the selection of vertebral augmentation therapy for osteoporotic vertebral compression fractures.

**Reference**

[1] Chinese Medical Association Osteoporosis and Bone Mineral Salt Diseases Branch. Guidelines for the diagnosis and treatment of primary osteoporosis (2022). Chinese Journal of Endocrinology and Metabolism. 2023 (05):377-406.

[2] Farooque M, Hillered, L., Holtz A, Olsson Y. Effects of moderate hypothermia on extracellular lactic acid and amino acids after severe compression injury of rat spinal cord. Journal of neurotrauma. 1997 Jan;14(1):63-69.

[3] Saracen A, Kotwica Z. Complications of percutaneous vertebroplasty: An analysis of 1100 procedures performed in 616 patients. Medicine (Baltimore). 2016 Jun;95(24):e3850.

[4] White Paper on Age-Related Visual Function and Eye Health Management. Chinese Journal of Optometry and Vision Science. 2022;24(1):1-9

[5] Ma X, Xue C, Wang X, Zhao Y, Meng W, Gao H, Pang Z, Liu X. Effect of multi-platform extended care on postoperative self-efficacy and quality of life in patients with osteoporotic vertebral compressive fracture. Am J Transl Res. 2021;13(6):6945-6951.

[6] Ballane G, Cauley JA, Luckey MM, El-Hajj Fuleihan G. Worldwide prevalence and incidence of osteoporotic vertebral fractures. Osteoporos Int. 2017 May;28(5):1531-1542.

[7] Dyreborg K, Sørensen MS, Flivik G, Solgaard S, Petersen MM. Preoperative BMD does not influence femoral stem subsidence of uncemented THA when the femoral T-score is > -2.5. Acta Orthop. 2021 Oct;92(5):538-543.

[8] Alsoof D, Anderson G, McDonald CL, Basques B, Kuris E, Daniels AH. Diagnosis and Management of Vertebral Compression Fracture. Am J Med. 2022 Jul;135(7):815-821.

[9] Klazen CA, Verhaar HJ, Lohle PN, Lampmann LE, Juttmann JR, Schoemaker MC, van Everdingen KJ, Muller AF, Mali WP, de Vries J. Clinical course of pain in acute osteoporotic vertebral compression fractures. J Vasc Interv Radiol. 2010 Sep;21(9):1405-1409.

[10] Osipov B, Emami AJ, Christiansen BA. Systemic Bone Loss After Fracture. Clin Rev Bone Miner Metab. 2018 Dec;16(4):116-130.

[11] Lau E, Ong K, Kurtz S, Schmier J, Edidin A. Mortality following the diagnosis of a vertebral compression fracture in the Medicare population. J Bone Joint Surg Am. 2008 Jul;90(7):1479-1486.

[12] Galibert P, Deramond H, Rosat P, Le Gars D. [Preliminary note on the treatment of vertebral angioma by percutaneous acrylic vertebroplasty]. Neurochirurgie. 1987;33(2):166-168.

[13] Shah LM, Jennings JW, Kirsch CFE, Hohenwalter EJ, Beaman FD, Cassidy RC, Johnson MM, Kendi AT, Lo SS, Reitman C, Sahgal A, Scheidt MJ, Schramm K, Wessell DE, Kransdorf MJ, Lorenz JM, Bykowski J. ACR Appropriateness Criteria(®) Management of Vertebral Compression Fractures. J Am Coll Radiol. 2018 Nov;15(11s):S347-s364.

[14] Osteoporosis Prevention and Rehabilitation Committee of the Chinese Society of Rehabilitation Medicine. Expert consensus on the diagnosis and treatment of osteoporotic vertebral compression fractures (2021 edition). Chinese Medical Journal. 2021 (41):3371-3379.

[15] Hollensteiner M, Botzenmayer M, Fürst D, Winkler M, Augat P, Sandriesser S, Schrödl F, Esterer B, Gabauer S, Püschel K, Schrempf A. Characterization of polyurethane-based synthetic vertebrae for spinal cement augmentation training. J Mater Sci Mater Med. 2018;29(10):153-153.

[16] Saracen A, Kotwica Z. Treatment of multiple osteoporotic vertebral compression fractures by percutaneous cement augmentation. Int Orthop. 2014 Nov;38(11):2309-2312.

[17] Khosla A, Diehn FE, Rad AE, Kallmes DF. Neither subendplate cement deposition nor cement leakage into the disk space during vertebroplasty significantly affects patient outcomes. Radiology. 2012 Jul;264(1):180-186.

[18] Yoshimura N, Muraki S, Oka H, Mabuchi A, En-Yo Y, Yoshida M, Saika A, Yoshida H, Suzuki T, Yamamoto S, Ishibashi H, Kawaguchi H, Nakamura K, Akune T. Prevalence of knee osteoarthritis, lumbar spondylosis, and osteoporosis in Japanese men and women: the research on osteoarthritis/osteoporosis against disability study. J Bone Miner Metab. 2009;27(5):620-628.

[19] Diamond TH, Bryant C, Browne L, Clark WA. Clinical outcomes after acute osteoporotic vertebral fractures: a 2-year non-randomised trial comparing percutaneous vertebroplasty with conservative therapy. Med J Aust. 2006 Feb 6;184(3):113-117.

[20] Diamond TH, Champion B, Clark WA. Management of acute osteoporotic vertebral fractures: a nonrandomized trial comparing percutaneous vertebroplasty with conservative therapy. Am J Med. 2003 Mar;114(4):257-265.

[21] Lee MJ, Dumonski M, Cahill P, Stanley T, Park D, Singh K. Percutaneous treatment of vertebral compression fractures: a meta-analysis of complications. Spine. 2009 May 15;34(11):1228-32.

[22] Nakano M, Hirano N, Ishihara H, Kawaguchi Y, Watanabe H, Matsuura K. Calcium phosphate cement-based vertebroplasty compared with conservative treatment for osteoporotic compression fractures: a matched case-control study. Journal of neurosurgery Spine. 2006 Feb;4(2):110-117.

[23] Wardlaw D, Cummings SR, Van Meirhaeghe J, Bastian L, Tillman JB, Ranstam J, Eastell R, Shabe P, Talmadge K, Boonen S. Efficacy and safety of balloon kyphoplasty compared with non-surgical care for vertebral compression fracture (FREE): a randomised controlled trial. Lancet. 2009 Mar 21;373(9668):1016-1024.

[24] Liu JT, Liao WJ, Tan WC, Lee JK, Liu CH, Chen YH, Lin TB. Balloon kyphoplasty versus vertebroplasty for treatment of osteoporotic vertebral compression fracture: a prospective, comparative, and randomized clinical study. Osteoporos Int. 2010 Feb;21(2):359-364.


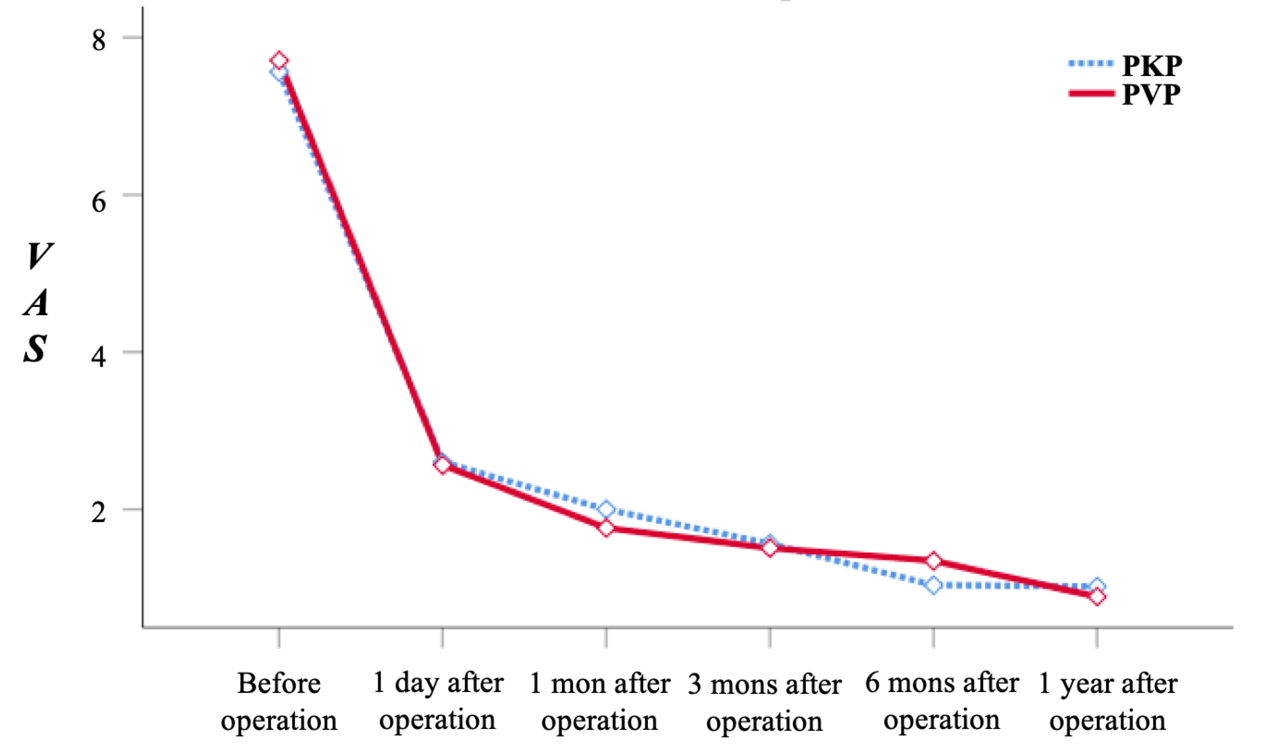
 Figure S1: Change trend of VAS scores of patients in two groups before and after surgery

Figure S2: Change trend of SF-36 scores of patients in two groups before and after surgery


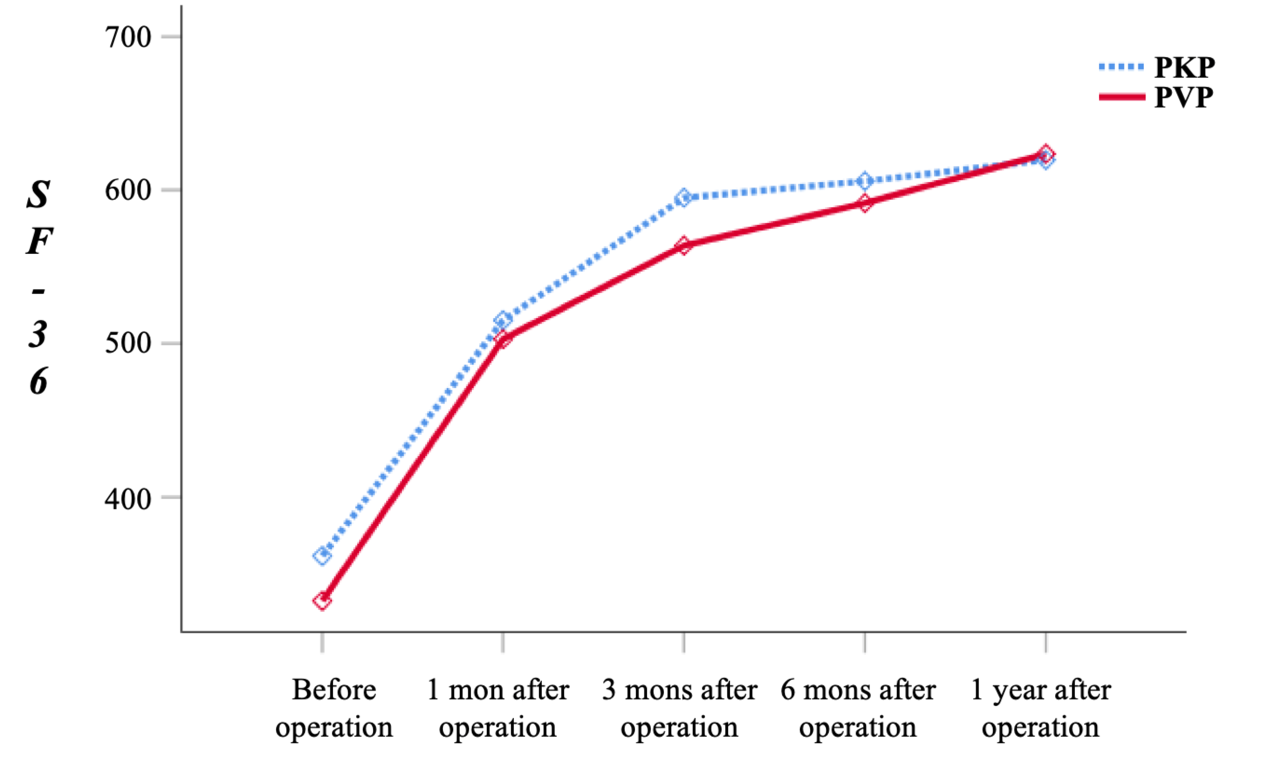

Supplement: zrad162_Supplementary_Data [file zrad162_supplementary_data.docx]
